# Screening and identification of critical transcription factors involved in the protection of cardiomyocytes against hydrogen peroxide-induced damage by Yixin-shu

**DOI:** 10.1038/s41598-017-10131-5

**Published:** 2017-10-24

**Authors:** Jingjing Zhang, Ya Geng, Feifei Guo, Fangbo Zhang, Mingwei Liu, Lei Song, Yuexiang Ma, Defeng Li, Yi Zhang, Haiyu Xu, Hongjun Yang

**Affiliations:** 1 Institute of Chinese Materia Medica, China Academy of Chinese Medical Sciences, Beijing, 100700 China; 2 College of Traditional Chinese Medicine, Shandong University of Traditional Chinese Medicine, Jinan, 250355 China; 3 State Key Laboratory of Proteomics, Beijing Proteome Research Center, Beijing Institute of Radiation Medicine, Beijing, 102206 China; 4 College of Traditional Chinese Medicine, Shandong University of Traditional Chinese Medicine, Jinan, 250355 China

## Abstract

Oxidative stress initiates harmful cellular responses, such as DNA damage and protein denaturation, triggering a series of cardiovascular disorders. Systematic investigations of the transcription factors (TFs) involved in oxidative stress can help reveal the underlying molecular mechanisms and facilitate the discovery of effective therapeutic targets in related diseases. In this study, an integrated strategy which integrated RNA-seq-based transcriptomics techniques and a newly developed concatenated tandem array of consensus TF response elements (catTFREs)-based proteomics approach and then combined with a network pharmacology analysis, was developed and this integrated strategy was used to investigate critical TFs in the protection of Yixin-shu (YXS), a standardized medical product used for ischaemic heart disease, against hydrogen peroxide (H_2_O_2_)-induced damage in cardiomyocytes. Importantly, YXS initiated biological process such as anti-apoptosis and DNA repair to protect cardiomyocytes from H_2_O_2_-induced damage. By using the integrated strategy, DNA-(apurinic or apyrimidinic site) lyase (Apex1), pre B-cell leukemia transcription factor 3 (Pbx3), and five other TFs with their functions involved in anti-oxidation, anti-apoptosis and DNA repair were identified. This study offers a new understanding of the mechanism underlying YXS-mediated protection against H_2_O_2_-induced oxidative stress in cardiomyocytes and reveals novel targets for oxidative stress-related diseases.

## Introduction

Reactive oxygen species (ROS) are a series of oxygen-based molecular species characterized by their high chemical reactivity, and the balance of the production and removal of ROS is vital in maintaining normal physiological processes^1,2^. During oxidative stress, ROS can induce a serious of biological changes such as DNA damage, lipid peroxidation and protein aggregation, which lead to cell death. Notably, oxidative stress-induced injury plays a vital role in myocardial ischaemia reperfusion (MI/R) injury by causing a series of complicated pathological changes such as DNA damage, mitochondrial dysfunction, and protein alteration, resulting in cell death and apoptosis^3^. Transcription factors (TFs) are proteins that bind to specific DNA sequences and regulate the transcription of DNA into RNA. Growing evidence has indicated that transcription factors act as critical regulators in many pathologic processes^4,5^. For example, sineoculis homeobox homolog 1 (Six1) decreased caspase-3 levels and inhibited tumor necrosis factor-related apoptosis^6^. Ring1 and YY1 binding protein (RYBP) induced tumour cell apoptosis by activating the activator protein 1 (AP-1) signalling pathway^7^. As an NF-κB family member, the transcription factor p65 (RELA) prevented JNK-mediated apoptosis in osteoclasts in response to the cytokine RANKL^8^. Recent studies have indicated that TFs may be critical regulators in oxidative stress injury^9,10^. The transcription factor GATA-4 (GATA4), a zinc finger-containing transcription factor, can protect cardiomyocytes from doxorubicin-induced apoptosis by recognizing the −266 GATA motif in the Bcl2 promoter^11^. Antioxidant and DNA repair genes could be activated by nuclear factor erythroid-derived-like 2 (Nrf-2) to promote cell survival when subjected to oxidative stress^9,10^. During ischaemia-reperfusion (IR), oxidative stress was enhanced by nuclear cardiac myosin light chain 2 (MYL2) through the up-regulation of NOX2 gene expression^12^.

Though TFs have been identified as important regulators in oxidative stress, direct large-scale profiling of TFs has remained a challenge, especially their quantitative evaluation. Currently, one method commonly used to estimate the abundance of TFs is the analysis of mRNA profiles^13,14^. However, the transcriptional activity of TFs cannot be directly inferred from mRNA profiling information. In addition, proteomics approaches can only detect a small portion of TFs, and their low abundance prevents the activity profiling of all TFs present in a cell, while the protein level of TFs is also insufficient to reflect their transcriptional activity^14^. Recently, a method for quantitatively evaluating the activity of TFs through large-scale profiling was established by building a DNA construct containing tandem transcription factor DNA response elements (catTFREs) with an affinity to specific TFs^15^. The catTFRE technology allows the direct identification and quantification of TF transcriptional activity in a high-throughput manner.

The catTFRE method provide a direct insight for the binding activity of transcription factors to a specific DNA sequence, which usually is the first step to activate downstream genes. The Illumina-based RNA-seq technology allows for a comprehensive examination of downstream gene expression^16,17^. Considering the binding activity of TFs to a specific DNA sequence and their downstream genes expression levels at the same time could reflect the activity and functions of the transcription factors more accurately and systematically. In this study, an integrated strategy which integrated TFs activity quantified by catTFRE method and their downstream genes by RNA-seq technology firstly and then combined with a network pharmacology analysis was developed and used to identify critical TFs in the protection of Yixin-shu (YXS), a Chinese standardized medical product used for the treatment of ischaemic heart disease, against H_2_O_2_-induced oxidative stress. YXS, containing seven herbs, *Ginseng*, *Ophiopogon japonicus*, Schisandra, Astragalus, Salvia, Chuanxiong, and Hawthorn, has been shown to maintain cardiac function by reducing mitochondrial-mediated apoptosis and oxidative stress injury^18^. However, the molecular mechanism by which YXS protects against oxidative stress remains unclear, especially on a TF level. Thus, an integrated strategy was developed in our research, and we hypothesized that this integrated strategy could be used to reveal critical TFs in the protection of YXS against H_2_O_2_-induced injury. Importantly, this integrated strategy could reduce the background interference to the minimum and find out the fundamental critical transcription factors in complicated biological process. Moreover, rather than focusing on a single or a few molecular pathways, a whole-transcriptome analysis and large-scale TF investigation, and then combined a network pharmacology approach help to ensure a comprehensive and accurate analysis.

## Materials and Methods

### Preparation of YXS Intestinal Absorption Liquid

Experimental Animal Center of Peking University Health Science Center, Beijing, China (certificate no. SCXK (Jing) 2009–0017) provided male Sprague Dawley rats weighting 220 ± 10 g. The experiment was approved by Committee on Animal Care and Use of Institute of Chinese Materia Medica, China Academy of Chinese Medical Sciences, and performed according to the approved guidelines. YXS capsule (Drug approval number: Z52020038) was provided by Guizhou Xinbang Pharmaceutical Co., Ltd(Guiyang, China). The powder of YXS capsule was dissolved in the ethanol (95%, v/v) and extracted for 2 hours under reflux. After filtered, the solution was rotary evaporated to dryness at 65 °C. Tyrode buffer solution (NaCl 8.00 g, KCl 0.28 g, MgCl_2_ 0.10 g, CaCl_2_ 0.20 g, NaHCO_3_ 1.00 g, NaH_2_PO_4_ 0.05 g, glucose 1.00 g, pH 7.4) was added to obtain YXS extraction solution (16%, w/v). After maintained in fasting conditions for 12 h, the rats were sacrificed and the intestines were collected. Four segments of intestines of 14 cm segments were generated and ligated to form a sac at one end after turned inside-out in Tyrode buffer solution at 0 °C. The Tyrode buffer in the sac was then exchanged with YXS solution (16%, w/v) at 37 °C with O_2_/CO_2_ (95%/5%). After 2 h, the solution in the sac was collected and filtered for the following experiments. The final concentration of YXS intestinal absorption liquid was 2 mg/mL, which was calculated according to the crude drug.

### Cell culture of H9c2 Cells and hiPS-CMs cells

Cell Resource Center of Peking Union Medical College in China offered H9c2 cells. The cells were cultured with culture medium containing penicillin (100 μg/ml), and streptomycin (100 μg/ml), high-glucose DMEM (Gibico, USA), and 10% v/v fetal bovine serum (Gibico, USA), at 37 °C in 5% CO_2_ incubator. Culture medium was replaced every 3 days. The cells were used for the following experiments after 80% cell confluence was achieved. The hiPS-CMs cells obtained from CELLAPYBIO (Cat# CA2001106, Beijing, China)^19^ and cultured according to its recommended procedures. The purchased hiPS-CMs cells were thawed and then cultured on the culture plates, which were pre-coated with 50 μL matrigel solutions (BD, 1:100) at 4 °C for 24 h. Then the cells were cultured in the incubator at 37 °C in 5% CO_2_ atmospheres. The medium was changed every 2 days. After nearly 5–7days, the hiPS-CM cells reached a stable cell status and used for the following experiment.

### Cell viability assays and Biochemical Analysis of SOD, MDA and T-AOC

The cell viability was performed by using 3-(4,5-dimethyl-2-thiazolyl)-2, 5-diphenyl-2H-tetrazolium bromide (MTT, Sigma M2128) assay. For the therapeutic effect, the cells were pre-treated with YXS intestinal absorption liquid of various concentrations (0, 7.81, 15.63, 31.25, 62.5 mg/ml) for 24 hours before H_2_O_2_ treatment for 1 hour. The safe concentration of YXS was investigated by treating H9c2 cells only with YXS for 24 hour. Trimetazidine (TMZ, sigma) with 10 μM was used as positive control. Then MTT solution was added to incubate for 3 hours and then treated with dimethyl sulfoxide (DMSO) before detected at 570 nm with a microplate reader (Molecular Devices, USA). As for the analysis of the total-antioxygen capacity (T-AOC), the total superoxide dismutase (T-SOD), and Glutaric dialdehyde (MDA), the treated cells were collected and then crushed through ultrasonic wave. Then investigation of T-AOC, T-SOD and MDA were performed according to the instruction of the detection kit with a microplate reader (Molecular Devices, USA), which were purchased from Nanjing Jiancheng (China).

### DCFH-DA analysis, JC-1 assay and Annexin V-PI staining

Intracellular reactive oxygen species (ROS) was assessed with the DCFH-DA probe (Nanjing Jiancheng, China). Briefly, 10 μM DCFH-DA was incubated with cell suspensions for 30 min and then residual probe was removed by washing with PBS. The cellular fluorescence intensities were measured on a fluorescence microplate reader (Molecular Devices, USA) with excitation and emission wavelengths set at 488 nm and 525 nm, respectively. Cell apoptosis was analyzed through annexin V-PI staining and JC-1 assay. After treatment, cell precipitate was collected through trypsinization and centrifugation. JC-1 dye (Beyotime, China) diluted with culture medium were added to the precipitation at 37 °C for 20 min. After washed twice, the cell suspension was detected with a FACStar Plus flow cytometer (Becton- Dickinson, USA). The mitochondrial membrane potential could be reflected by the Mean FL2 fluorescence intensity. Annexin V-FITC and PI staining was applied to investigate the cell apoptotic rate by using an Annexin V-FITC Apoptosis Detection Kit. Briefly, after treatment, 500 *μ*L binding buffer was added and then 5 *μ*L Annexin V and 5 *μ*L propidium iodide was loaded in the dark at room temperature for 10 min. Photos were taken under a confocal microscope.

### Immunofluorescence staining

For the immunofluorescence staining, 10% polyformaldehyde was applied to fix the samples, followed by permeabilizing with 0.5% Triton X-100 for 30 min. After washed with PBS, the samples were treated with 10% donkey serum to block background for 60 min. Next, the primary antibody including cleaved Caspase-3 (Proteintech 25546-1-AP, 1:500), APEX1 (Proteintech 10203-1, 1:100), PBX3 (Proteintech 12571-1-AP, 1:100), was loaded at 4 °C for 24 h, followed by rhodamine phalloidin (PHDR1, cytoskeleton) and a secondary anti-body (ab 150073, 1: 200) incubation in the dark at 37 °C for 60 min. Finally, the samples were treated with 4,6-diamidino-2-phenylindole (DAPI; Sigma, USA) for 5 min before photos were taken under the confocal microscope (LSM510; Zeiss).

### RNA extraction, library preparation and sequencing

Three biological replicates were used for the RNA-seq experiment. After treatment, total RNA was extracted using TRIzol Reagent (Cat#15596-018, Life Technologies, USA) according to the manufacturer’s instructions. An RNA Nano 6000 Assay Kit was used to detect the RNA integrity on a Bioanalyzer 2100 system (Agilent Technologies, CA, USA). Then, 1 μg of RNA was used to construct a cDNA library with a NEBNext® Ultra™ RNA Library Prep Kit for Illumina® (NEB, USA). Briefly, poly-T oligo-conjugated magnetic beads were used to purify mRNA, and then the purified mRNA was cleaved into fragments by divalent cations before the synthesis of first-strand cDNA. Next, second-strand cDNA was synthesized using DNA Polymerase I and RNase H. Ligation of the adapters and adenylation of the 3′ ends of DNA fragments was completed before PCR was used to amplify the cDNA template. A TruSeq PE150 Cluster Kit v3-cBot-HS (Illumina) was used to perform the clustering of the index-coded samples on a cBot Cluster Generation System. An Illumina HiSeq. 4000 platform was used to sequence the library, and 150 bp paired-end reads were produced. The library construction and Illumina sequencing were performed at Novogene Bioinformatics Technology Co., Ltd. (Beijing, China), and the raw-sequence read data of the whole experiment were uploaded to https://www.ncbi.nlm.nih.gov/bioproject/PRJNA361184.

The rat reference genome (ensemble release 83) was used for read mapping for the subsequent transcript assembly and quantification, and HTseq v0.6.1 was used to count the number of reads mapped to each gene. To estimate gene expression levels, FPKM was calculated and represented as the number of fragments per kilobase of transcript sequence per millions of base pairs sequenced. The differential expression analysis was performed using edgeR software^20^. FDR correction for multiple testing was applied to discover differentially expressed genes (FDR < 0.05). ClueGO^21^ was used for the functional annotation of the RNA-seq data, which was further visualized with Cytoscape v3.4.0^22^.

### Quantifications of transcription factor activity by catTFREs method

Nuclear extracts of the samples were obtained using nuclear extract prep kits (Thermo Fisher) according to the manufacturer’s protocol. Biotinylated DNA was pre-immobilized on Dynabeads before the nuclear extract was added and incubated with the Dynabeads. EDTA/EGTA and NaCl were then added to this mixture to obtain a final concentration of 1 mM and 200–250 mM, respectively, and the mixture was incubated at 4 °C for 2 h. The supernatant was removed, and the Dynabeads were washed with NETN [0.5 mM EDTA, 100 mM NaCl, 20 mM Tris-HCl, and 0.5% (vol/vol) Nonidet P-40] twice before the beads were digested overnight with trypsin. The tryptic peptides were analysed by an LTQ-Orbitrap Velos instrument (Thermo) after separation on a C18 column (75 μm inner-diameters, 360 μm outer-diameter × 10 cm, 3 μm C18), with the flow rate set at 350 nL/min. The MS conditions were set as follows: A nano-spray ion source with a spray voltage of 1800 V, with no sheath gas flow and with the ion transfer tube at 350 °C, was used. A data-dependent mode was used in the mass spectrometer with a survey scan from m/z 375 to 1600 with a resolution of 60,000 at m/z 400. Collision-induced dissociation with normalized collision energy of 35% and an activation time of 5 ms was applied to acquire the 50 most intense peaks with a charge state no less than 2.

LC-MS/MS analyses were conducted on an Easy-nLC 1000 liquid chromatography system (Thermo), which was coupled to an Orbitrap Fusion through a nano-electrospray ion source (Thermo). The obtained tryptic peptides were isolated on a homemade 100 µm ID × 10 cm column (C18, 1.9 µm, 120 Å, Dr. Maisch GmbH) at 500 nL/min with a linear 5–35% acetonitrile gradient after being eluted from a 360-um ID × 2 cm, C18 trap column. Survey scans were acquired after the accumulation of 5e5 ions in the Orbitrap for m/z 300–1400 using a resolution of 120,000 at m/z 200. After the fragmentation was conducted in top-speed data-dependent mode at normalized collision energy of 32% in the HCD cell, the fragmented ions were transferred into the ion trap analyser with the AGC target at 5e3 and maximum injection time at 35 ms. The dynamic exclusion of previously acquired precursor ions was set to be 18 s.

The rat protein RefSeq database in Proteome Discoverer 1.4.0.288 was used to analyse the spectral data. Mascot (version 2.3.01, Matrix Science) was used to ensure a false discovery rate lower than 1%. The mass tolerance for the precursor was set to be 20 ppm, while the tolerance for product ions was set at 0.5 Da. Specifically, acetyl (N terminus) and oxidation (Met) were set as variable modifications and carbamidomethyl (Cys) as a fixed modification, and 2 missed cleavage sites for trypsin were permitted. An intensity-based absolute quantification method was applied to analyse the protein level. Briefly, the number of theoretical peptides was calculated by in silico protein digestion with a PERL script, and the numbers of peptides between 6 and 30 amino acids were added, while the missed cleavages were ignored. The iBAQ intensities were acquired by normalizing the protein intensities to the number of theoretical peptides

### Algorithm of critical TFs detection and regulatory network construction

Based on expression level and regulatory relationships of TFs and target genes, critical TFs were identified as TFs whose target genes expression level was differentially expressed accordingly. According to consistency of transcriptional activity of TF and expression level of downstream targets, critical TFs were classified into two categories: activators and repressors; in two conditions, change of transcription activity of former and downstream target gene expression is consistent which suggested activator may activated transcription of targets, and change of the latter is inconsistent which suggested repressors may repressed target transcription. Permutation test was applied to test whether there is statistically significant change of target genes expression using whole transcriptome as null distribution to compare, and only the TFs with significantly differential expressing of target genes were considered as critical TFs. For a group of targets, T is a vector which includes fold change of each target expression in two conditions (e.g. model vs. control or YXS vs. model). G is a vector which presents fold change of all genes expression in two conditions. Permutation test is applied to test whether there is significant difference between means of T and G (null distribution). A group of targets was significantly up-regulated, if mean of T is larger than G with significant difference; otherwise this group of targets was significantly down-regulated. Gene regulatory relationships were retrieved from the mouse gene regulatory network of CellNet by vertebrate homology mapping to rat^23^. Critical TFs and their downstream target genes regulatory relationships were used to construct TF-target regulatory network. Among target genes, only differentially expressed gene were included. Co-occurring TFs and target were colored as pink node by comparing regulatory network of model (model vs control) and after YXS pretreatment (YXS vs model).

### Prediction of Potential targets for chemical compounds

Chemical compounds of YXS were obtained from a published literature^24^ and then were submitted to BATMAN-TCM^25^ to discover TCM therapeutic mechanism. The potential targets of 83 chemical compounds were obtained by the retrieval in the integrated known drug-target interaction dataset and the similarity-based target prediction method which ranks potential drug-target interactions based on their similarity to the known drug-target interactions, respectively^26^.

### Statistical analysis

The data obtained in this research was analyzed with SPSS V17.0 (one-way ANOVA, LSD, p < 0.05) and the results are shown as mean ± standard deviation (SD). The significant difference was set at P values, *P < 0.05 versus control, ^#^P < 0.05 versus model.

## Results

### YXS protects against H_2_O_2_-induced damage through increased cell viability and decreased cell apoptosis

**Figure 1:**
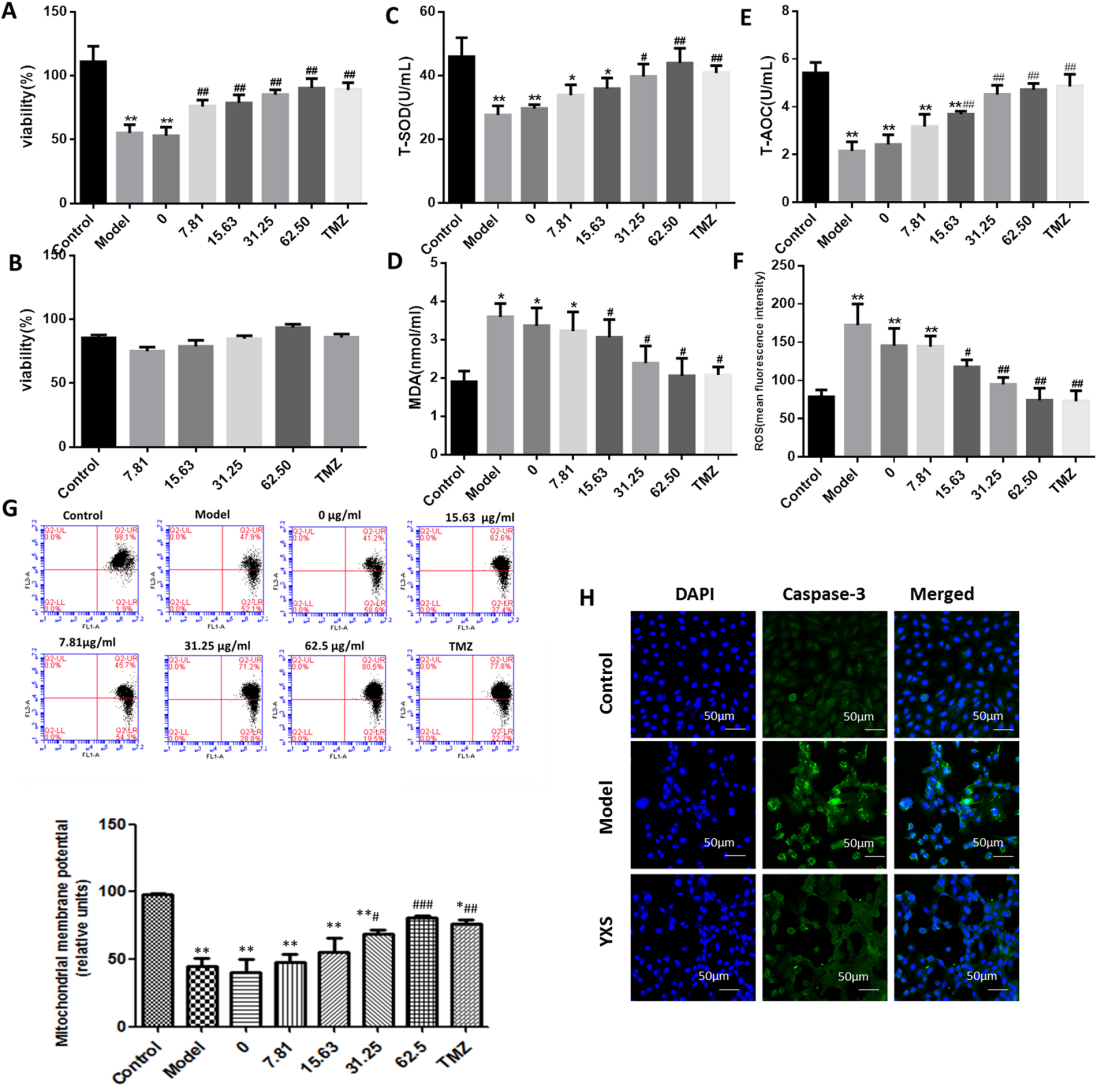
The protective effect of YXS against H_2_O_2_-induced damage. (**A**) Cell viability. (**B**) Viability of cells treated only with YXS. (**C**) Total superoxide dismutase activity (T-SOD). (**D**) Glutaraldehyde (MDA). (**E**) Total antioxygen capacity (T-AOC). (**F**) Reactive oxygen species (ROS) accumulation. (**G**) Mitochondrial membrane potential. (**H**) Cleaved caspase-3 visualized by immunofluorescence staining. Cleaved caspase-3 is stained red, while the nucleus is stained blue. Scale bar: 50 µm. Control: H9c2 cells alone; Model: H9c2 cells treated with H_2_O_2_ for 1 h; YXS: H9c2 cells pre-treated with YXS for 24 h and then with H_2_O_2_ for 1 h. Cleaved caspase-3: green; nucleus: blue. Scale bar: 50 µm. The data are presented as the mean ± SD *P < 0.05 compared to the control group; ^#^ *P* < 0.05 compared to the model group.

To investigate the effect of YXS on H_2_O_2_-induced damage, cell viability, antioxidant enzymes, ROS levels and cell apoptosis were examined accordingly (Fig. 1). Cell viability was remarkably decreased to 51.64 ± 5.33% by H_2_O_2_ when compared to the control cells, while YXS increased cell viability in a dose-dependent manner, and the cell viability reached 89.21 ± 6.17% with 62.5 μg/ml YXS treatment (Fig. 1A). YXS alone did not affect cell viability (Fig. 1B). The activity of oxidative stress-related biochemical enzymes such as T-AOC and T-SOD increased and metabolites such as MDA decreased in a dose-dependent manner with the increasing concentrations of YXS (Fig. 1C,D and E). ROS generation and loss of the mitochondrial membrane potential are thought to be important processes in oxidative stress. YXS decreased ROS generation after H_2_O_2_ treatment, as analysed by the H2DCFDA probe (Fig. 1F). Furthermore, the loss of mitochondrial membrane potential is a characteristic of early-stage apoptosis. Mitochondrial membrane potential loss after exposure to H_2_O_2_ was obviously improved by YXS, indicating that YXS treatment resulted in a decrease in mitochondria-mediated apoptosis. Moreover, immunofluorescence staining of cleaved caspase-3 also indicated that YXS decreased cell apoptosis at the molecular level (Fig. 1G). Thus, an obvious protective effect of YXS was observed in H9c2 cells in response to H_2_O_2_, as indicated by the increased cell viability and antioxidant enzymes level such as SOD and T-AOC while  decreased MDA production, ROS generation and cell apoptosis.

### Apoptosis-related TFs played a vital role in H_2_O_2_-induced damage as analysed by catTFRE methods

**Figure 2:**
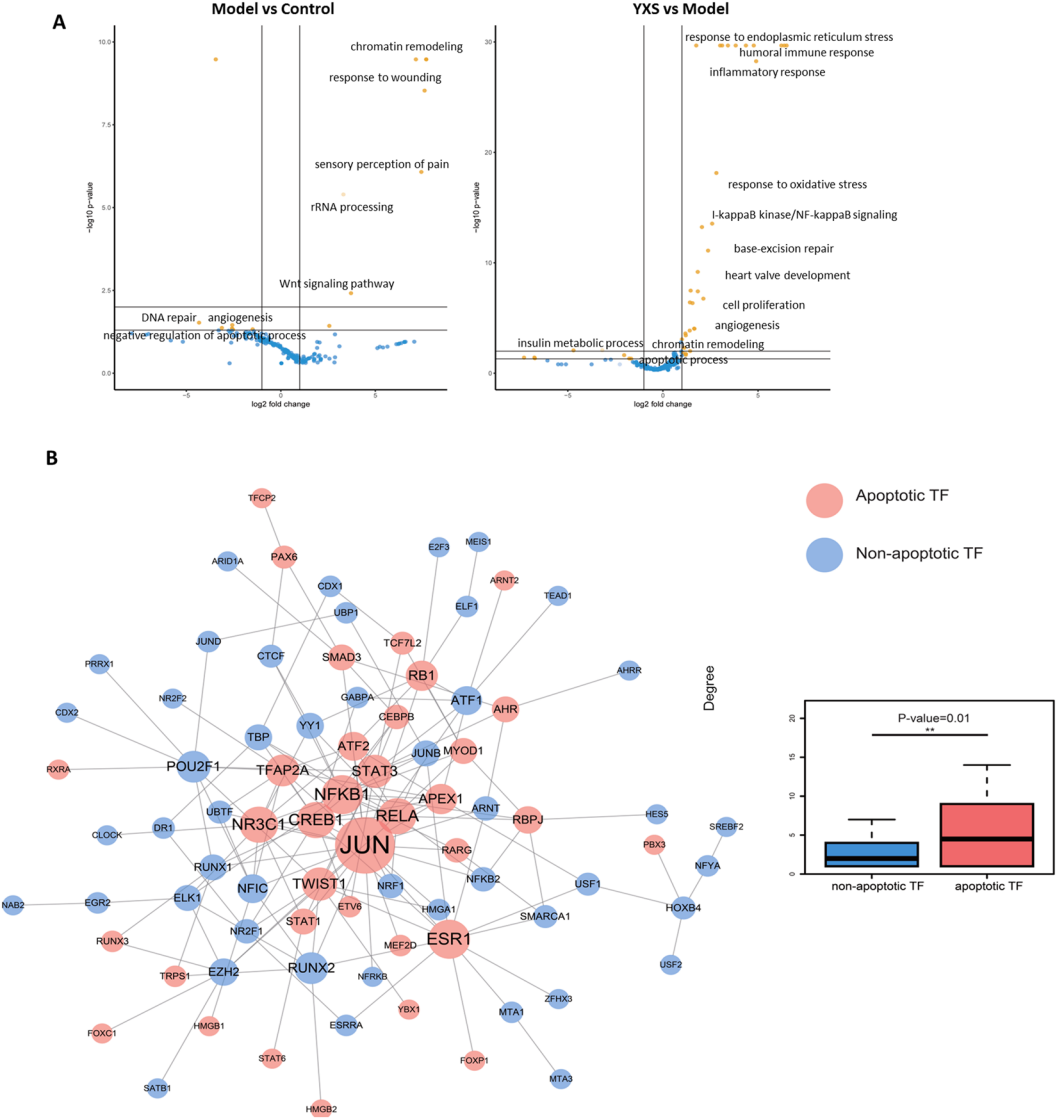
The large-scale quantitative profiling of transcription factor (TF) activity in H9c2 cells in response to H_2_O_2_-induced oxidative stress. (**A**) The molecular functions of the significantly altered TFs (P < 0.05) are indicated with yellow dots, and the blue dots represent TFs that showed no differences. (**B**) TF regulatory network and boxplots of the network degree for apoptotic TFs and non-apoptotic TFs in the regulatory network.

To obtain a quantitative profile of TF activity, the catTFRE method was used to detect TF activity immediately after H_2_O_2_ treatment, and the data were analysed using an intensity-based absolute quantification (iBAQ) approach. As shown in Table S1, 205 TFs were identified to be altered upon the induction of oxidative stress by H_2_O_2_, with 57 activated TFs (fold change > 2) and 51 repressed TFs (fold change < 0.5) identified in the H_2_O_2_-treated group compared with the control group (Table S1). By contrast, YXS activated 34 TFs and repressed 36 TFs (fold change < 0.5) when compared to the model group. The analysis of TF biological functions further revealed that H_2_O_2_ treatment activated TFs (yellow dots in Fig. 2A) that participate in chromatin remodelling, response to wounding, and rRNA processing and repressed TFs (blue dots in Fig. 2A) involved in DNA repair, angiogenesis, and negative regulation of apoptotic processes. In contrast to the model group, YXS activated TFs involved in response to endoplasmic reticulum stress, inflammatory response, cell proliferation, base-excision repair and angiogenesis, while it repressed TFs involved in apoptotic processes, chromatin remodelling and insulin metabolic processes. Further analysis of the 205 TFs revealed that there were 48 TFs involved in cell apoptosis based on Gene Ontology terms related to apoptosis (GO:0006915, GO:0043065 and GO:0043065)^27^, and the apoptosis-related TFs had a significantly larger network degree than the non-apoptosis-related TFs (5.85 vs 2.83, p = 0.0128 by Mann-Whitney U test, Fig. 2B), indicating that these apoptosis-related TFs occupied an important position in the TF regulatory network and that cell apoptosis played a vital role in H_2_O_2_-induced oxidative stress.

### Whole-genome transcriptome profiling of gene expression by RNA-seq technology

**Figure 3:**
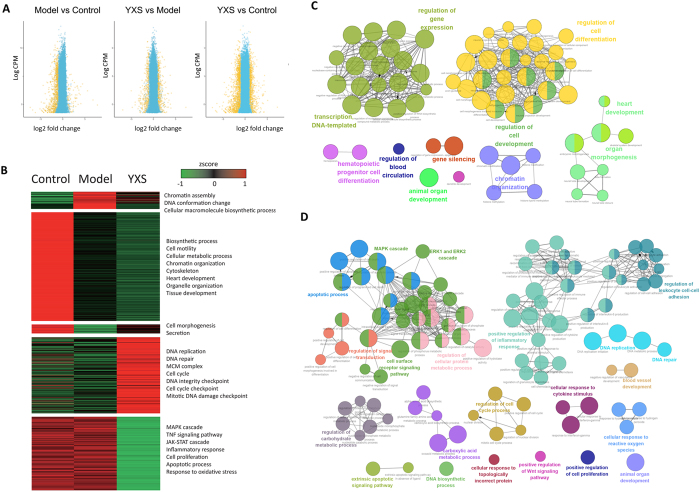
Gene expression in H9c2 cells in response to H_2_O_2_-induced oxidative stress. (**A**) The majority of the differentially expressed (DE) genes were suppressed by H_2_O_2_ treatment, whereas YXS could recover the gene expression profile almost to the level of the control group. Yellow dots indicate differentially expressed genes, and blue dots represent genes with no significant difference. (**B**) Hierarchical clustering of differentially expressed genes in response to H_2_O_2_ treatment. (**C** and **D**) Over-represented GO terms for differentially expressed (DE) genes obtained from RNA-seq technology in the H_2_O_2_-induced model group compared with the control group (**C**) and in the cells pre-treated with YXS compared with the model group (**D**) by ClueGO.

RNA-seq technology was used to investigate the gene expression profile of YXS-mediated protection against H_2_O_2_-induced oxidative stress. This method revealed that the general repressed gene expression in response to H_2_O_2_ could be rescued by YXS (Fig. 3A). Specifically, there were 584 significantly down-regulated genes and 52 up-regulated genes in the H_2_O_2_-treated model cells. In contrast to the model group, the gene expression profile was reversed to some extent by YXS, as indicated by 281 up-regulated genes and 246 down-regulated genes (Table S2). As shown in Fig. 3B, the differentially expressed genes were clustered, and the functional annotation of these genes was analysed by DAVID^27^. This analysis revealed that biological processes such as chromatin assembly and DNA conformation change were activated and that biosynthetic processes, cell mobility, cytoskeleton, and heart development were repressed when comparing H_2_O_2_-treated and untreated groups, indicating that damage may be caused to the cell cytoskeleton and DNA structure. Interestingly, in contrast to the model group, YXS up-regulated DNA repair-related processes such as DNA integrity checkpoints, cell cycle checkpoints, mitotic DNA damage checkpoints and the MCM complex, while it down-regulated apoptosis-related processes such as chromatin assembly, DNA conformation change, apoptotic processes, response to oxidative stress, the inflammatory response, the TNF signalling pathway, and the MAPK and JAK-STAT cascades. The differential gene expression patterns indicated that YXS helps H9c2 cells establish a process to resist external stimuli by down-regulating biological processes such as apoptosis and cell death while up-regulating genes involved in DNA repair, cell cycle and DNA damage checkpoints.

To further explore the molecular mechanism, the over-represented Gene Ontology (GO) terms were determined (Fig. 3C and D). When comparing the H_2_O_2_-treated and untreated groups, the differentially expressed genes were clustered into two major over-represented GO terms, gene expression-related terms and cell differentiation and development-related terms (Fig. 3C). Specifically, the gene expression-related terms included “transcription DNA-templated”, “regulation of gene expression”, and “gene silencing”, while the cell differentiation and development-related GO terms included “hematopoietic progenitor cell differentiation” “animal organ development” “regulation of cell differentiation”, and “heart development”. In contrast to the model group, over-represented cell growth-related GO terms, such as “regulation of cell cycle process”, “positive regulation of cell proliferation” and “DNA replication”, and “DNA biosynthetic process”, and metabolic-related GO terms, such as “regulation of carbohydrate metabolic process”, and “regulation of cellular protein metabolic process”, were observed after YXS treatment (Fig. 3D). In addition, GO terms such as “MAPK cascade”, “ERK1 and ERK2 cascade”, “positive regulation of Wnt signaling pathway”, “positive regulation of inflammatory response”, and “cellular response to cytokine stimulus” were also shown. Notably, apoptosis-related terms such as “apoptotic process” and “extrinsic apoptotic signaling pathway” and DNA repair-related terms such as “DNA repair” appeared when comparing the YXS and model groups. Taken together, these results indicated that to reduce H_2_O_2_-induced damage, a protective process was established by YXS through the down-regulation of apoptosis processes and cell proliferation and the up-regulation of DNA repair and cell cycle processes.

### Critical TFs preliminarily identified by integrating the alterations in TF activity and the expression of their downstream target genes

**Figure 4:**
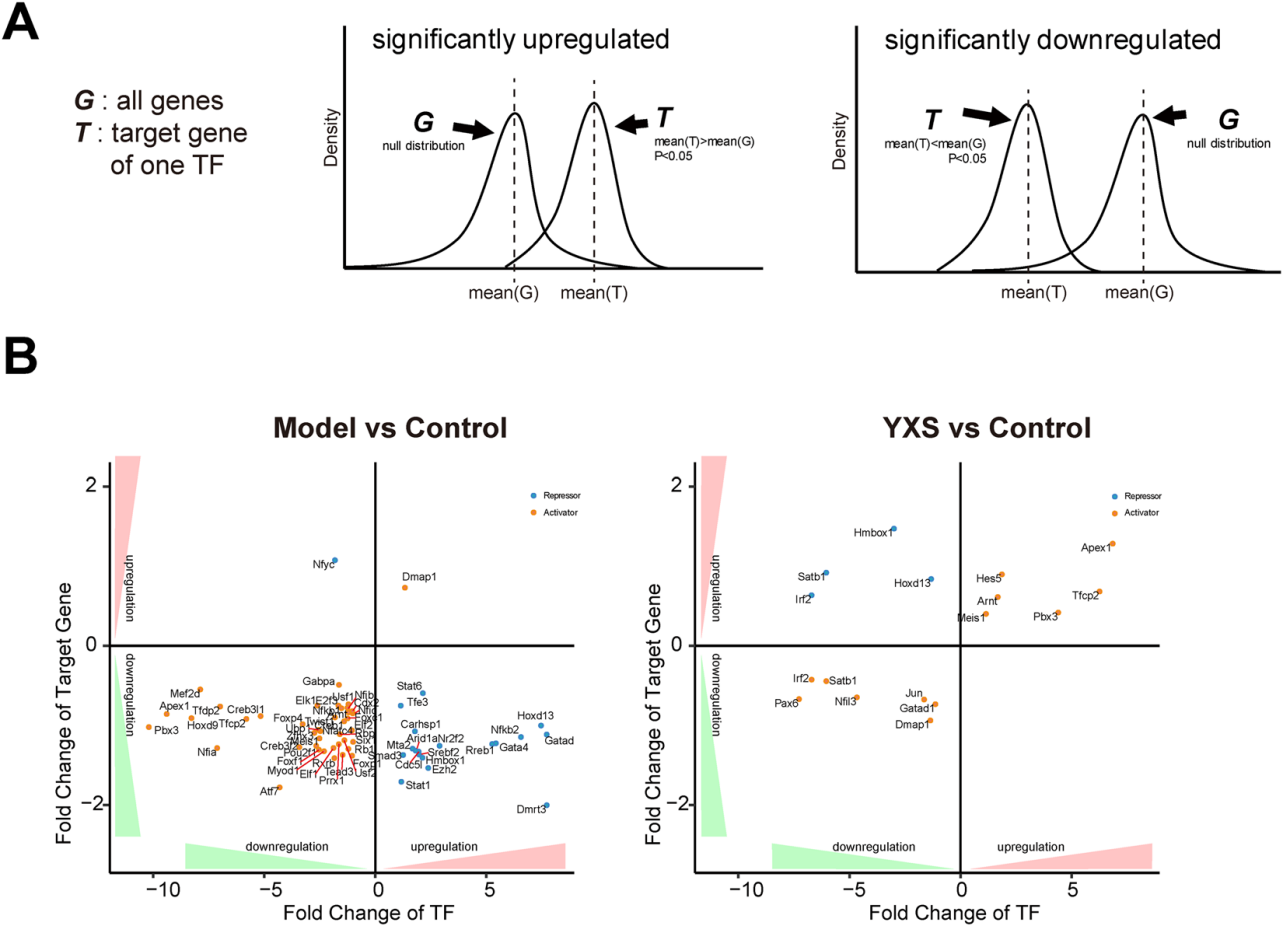
The correlation between critical TFs and their target genes in response to H_2_O_2_-induced oxidative stress, based on the direction of TF regulation and the expression level of TFs’ target genes. Yellow nodes refer to activators; blue nodes refer to repressors.

**Figure 5:**
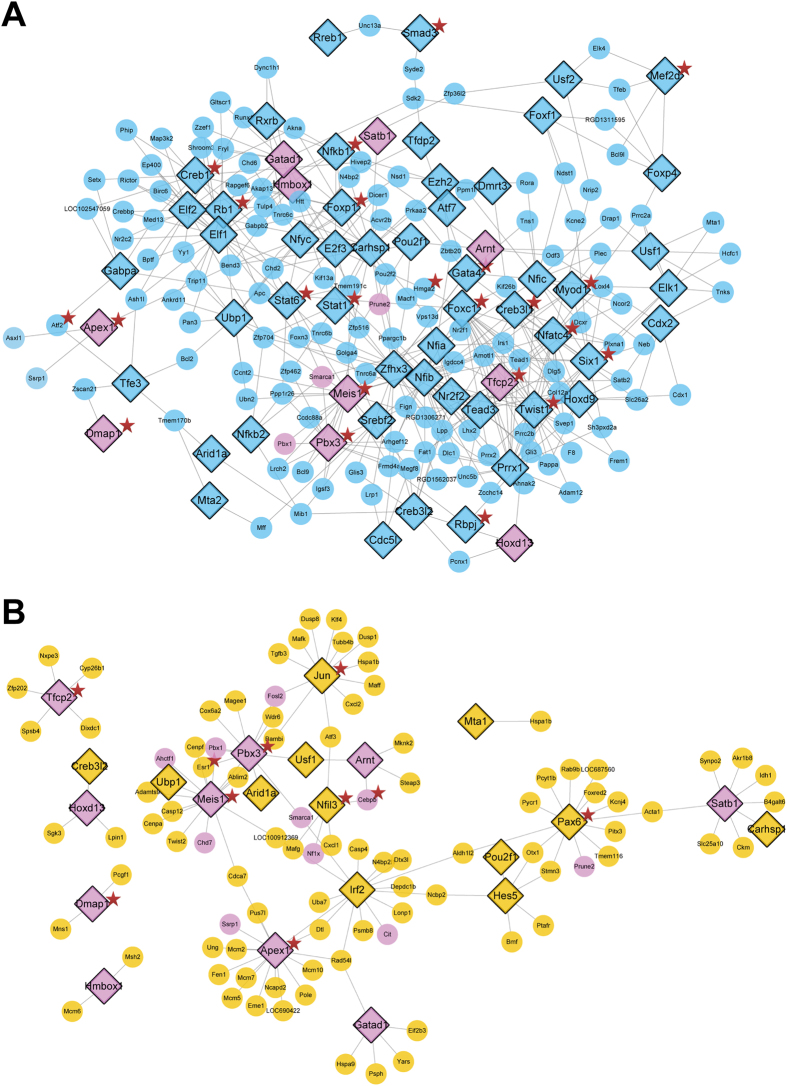
The regulatory network of transcription factors (TFs) and their differentially expressed target genes (TG) as determined by TF-TG regulatory relationships from CellNet. (**A**) The TF-TG regulatory network of H9c2 cells treated with H_2_O_2_ in comparison to the control cells. Blue indicates TFs changed only in the model group compared to the control group. (**B**) The TF-TG regulatory network of H9c2 cells pre-treated with YXS in comparison to the model group. Yellow indicates TFs altered only in the YXS-treated cells. Pink indicates TFs that were co-regulated in both the model group compared with the control and the YXS group compared with the model group. A star marker was added to indicate TFs or targets that are involved in apoptotic processes.

To identify critical TFs in the H_2_O_2_-induced injury model, an algorithm was developed based on the transcriptional activity of TFs and the expression level of target genes. The critical TFs were divided into two classes, activators and repressors, based on the direction of TF regulation and the expression of target genes. The correlations between critical TFs and their target genes in response to H_2_O_2_ are shown in Fig. 4 based on the direction of TF regulation and the expression of target genes. Notably, most activators were repressed and most repressors were activated in response to H_2_O_2_ treatment, which was in agreement with the global repressed gene expression profile in the model group (Fig. 3A). After YXS treatment, 6 activators were activated, and 4 repressors were repressed, suggesting a rescue effect of YXS in the prevention of H_2_O_2_-induced damage on a transcription factor level. To comprehensively elucidate the TF-target relationship, a network of TF-target regulatory events in different situations is shown in Fig. 5. Briefly, 63 TFs (blue nodes) were significantly altered in the model group when compared with the control group (Fig. 5A). In contrast to the model group, only 22 TFs were significantly altered by YXS, including 12 TFs (yellow nodes) regulated only in the YXS group compared with the model group and 10 TFs (pink nodes, co-regulated TFs) regulated in both the YXS group compared with the model group and the model group compared with the control group. Thus, through integrating TF activity and the expression of their downstream genes, these 10 co-regulated TFs were preliminarily identified as critical transcription factors in oxidative stress.

### Further identification of critical TFs through a network of chemical components of YXS and their potential targets

**Figure 6:**
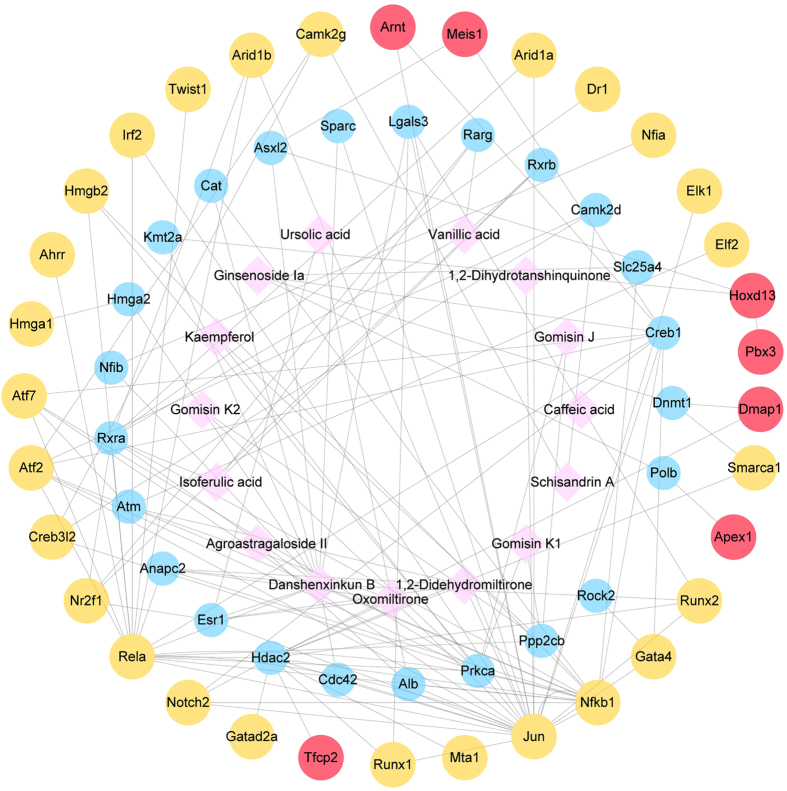
Interaction network of chemical compounds of YXS and their potential targets. There are two types of interactions in this network, interactions between chemical compounds of YXS (pink) and critical TFs (blue) are potential drug-target interactions, and interactions between co-activators (blue) and TFs (yellow and red) are high confidence protein-protein interactions (confidence score ≥0.5) from STRING. Red nodes represent further identified critical TFs.

A network consisting of the potential targets (24 co-activators and 33 critical TFs) and constituent chemical compounds (15 compounds) was constructed to demonstrate the connection between these critical TFs in oxidative stress and YXS. This network revealed that 24 transcriptional co-activators were co-expressed with their interacting critical TFs after YXS treatment (Fig. 6). Among these 33 critical TFs, 7 TFs were found to interact with chemical components of YXS through their transcriptional co-activators. These factors including DNA-(apurinic or apyrimidinic site) lyase (Apex1), pre B-cell leukemia transcription factor 3 (Pbx3), alpha-globin transcription factor CP2 (Tfcp2), homeobox protein Hox-D13 (Hoxd13), DNA methyltransferase 1-associated protein 1 (Dmap1), aryl hydrocarbon receptor nuclear translocator (Arnt), and homeobox protein Meis1 (Meis1), were important in the YXS-mediated protection against oxidative stress.

### Apex1 and Pbx3 activity verification and their target gene expression in H9c2 cells

**Figure 7:**
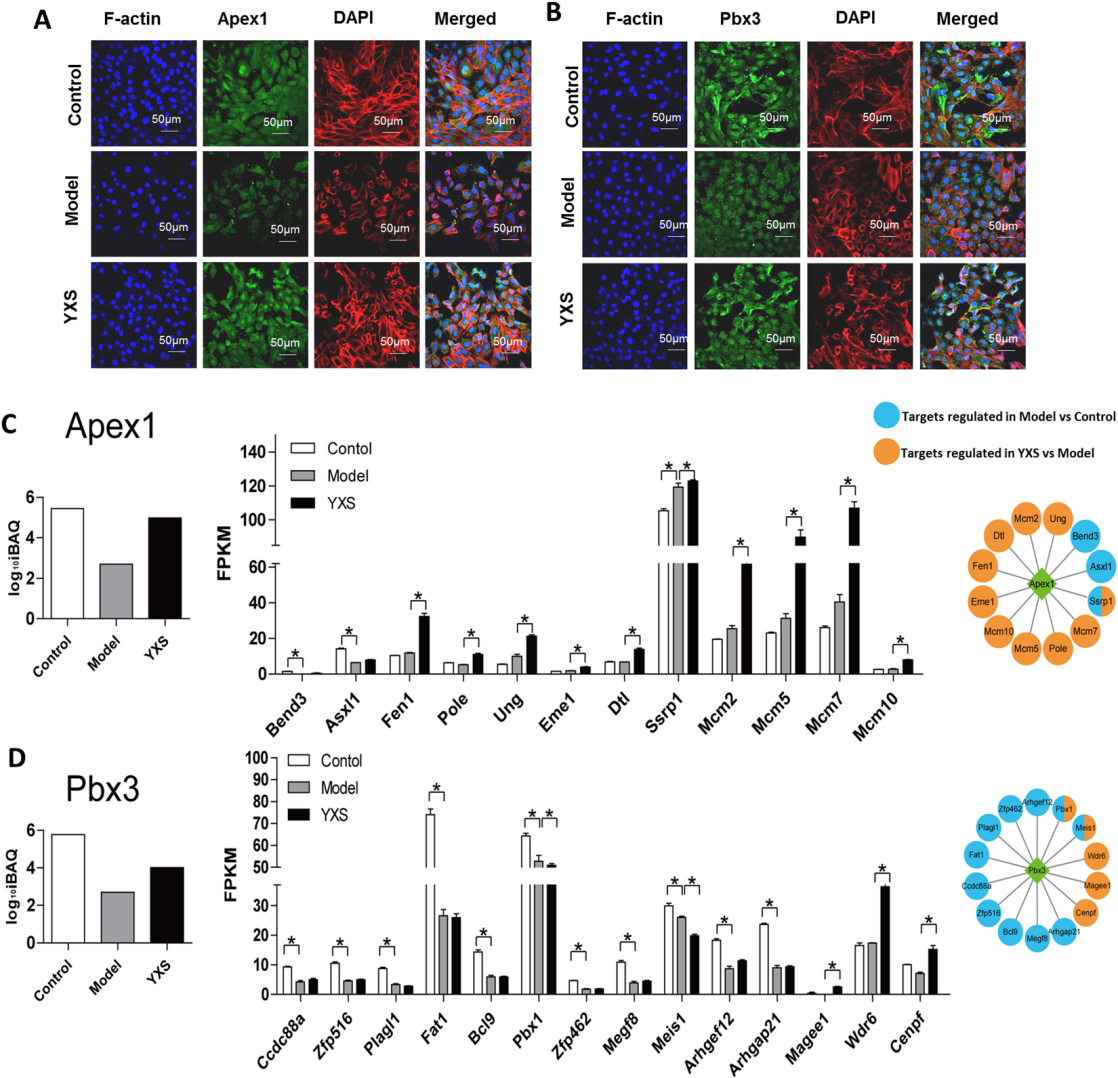
Transcriptional activity of Apex1 and Pbx3 and their downstream genes in H9c2 cells in response to H_2_O_2_-induced oxidative stress. The transcriptional activity of (**A**) Apex1 and (**B**) Pbx3 verified by immunofluorescence staining. Apex1 and Pbx3: green; F-actin: red, nucleus: blue. Scale bar: 50 µm. The target gene expression of (**C**) Apex1 and (**D**) Pbx3. The target genes of Apex1 and Pbx3 were all positively regulated by the corresponding TFs. Significantly different expression of a target gene is marked as P < 0.05.

Apex1 plays a central role in the response to oxidative stress, and its major role is in DNA repair and cell redox homeostasis regulation^28,29^. Pbx3 belongs to a family of TALE (three amino acid loop extension) class homeodomain transcription factors, which are involved in developmental gene expression^30^. To further verify the transcriptional activity of Apex1 and Pbx3, immunofluorescence staining was performed. The transcriptional activity of Apex1 and Pbx3 was suppressed, as indicated by a decrease in the nuclear staining of Apex1 and Pbx3 in the H_2_O_2_-treated group (Fig. 7A,B). However, the decreased nuclear staining was rescued by YXS treatment, as indicated by a stronger nuclear staining of Apex1 and Pbx3 in the YXS group than in the model group, demonstrating higher Apex1 and Pbx3 transcriptional activity in the YXS group. This result was in agreement with the results from the catTFRE method. The expression of their downstream genes was analysed in Fig. 7C and D. Among the genes positively regulated by Apex1, the expression of Asxl1 and Bend3 was significantly decreased in the model group when compared with the control group, while the expression levels of Fen1, Pole, Ung, Eme1, Dtl, Ssrp1, Mcm2, Mcm5, Mcm7 and Mcm10 were significantly increased in the YXS group when compared with the model group, indicating that the repression of Apex1 by H_2_O_2_ treatment was reversed by YXS (Fig. 6C). The expression of genes downstream of Pbx3 was also consistent with the alteration of Pbx3 activity, in that the down-regulation of Arhgef12, Magee1, and Cenpf by H_2_O_2_ was reversed in the YXS group (Fig. 6C). In addition, the gene expression of Ccdc88a, Zfp516, Plagl1, Fat1, Bcl9. Pbx1, Zfp462, Megf8, and Arhgap21 was significantly down-regulated, while the gene expression of Magee1, Wdr6 and Cenpf was significantly up-regulated in YXS-treated group (Fig. 7D). The expression of target genes further confirmed the alteration of Pbx3 activity as indicated by the catTFRE method.

### Further verification of the transcriptional activity of Apex1 and Pbx3 in hiPS-CM cells

**Figure 8:**
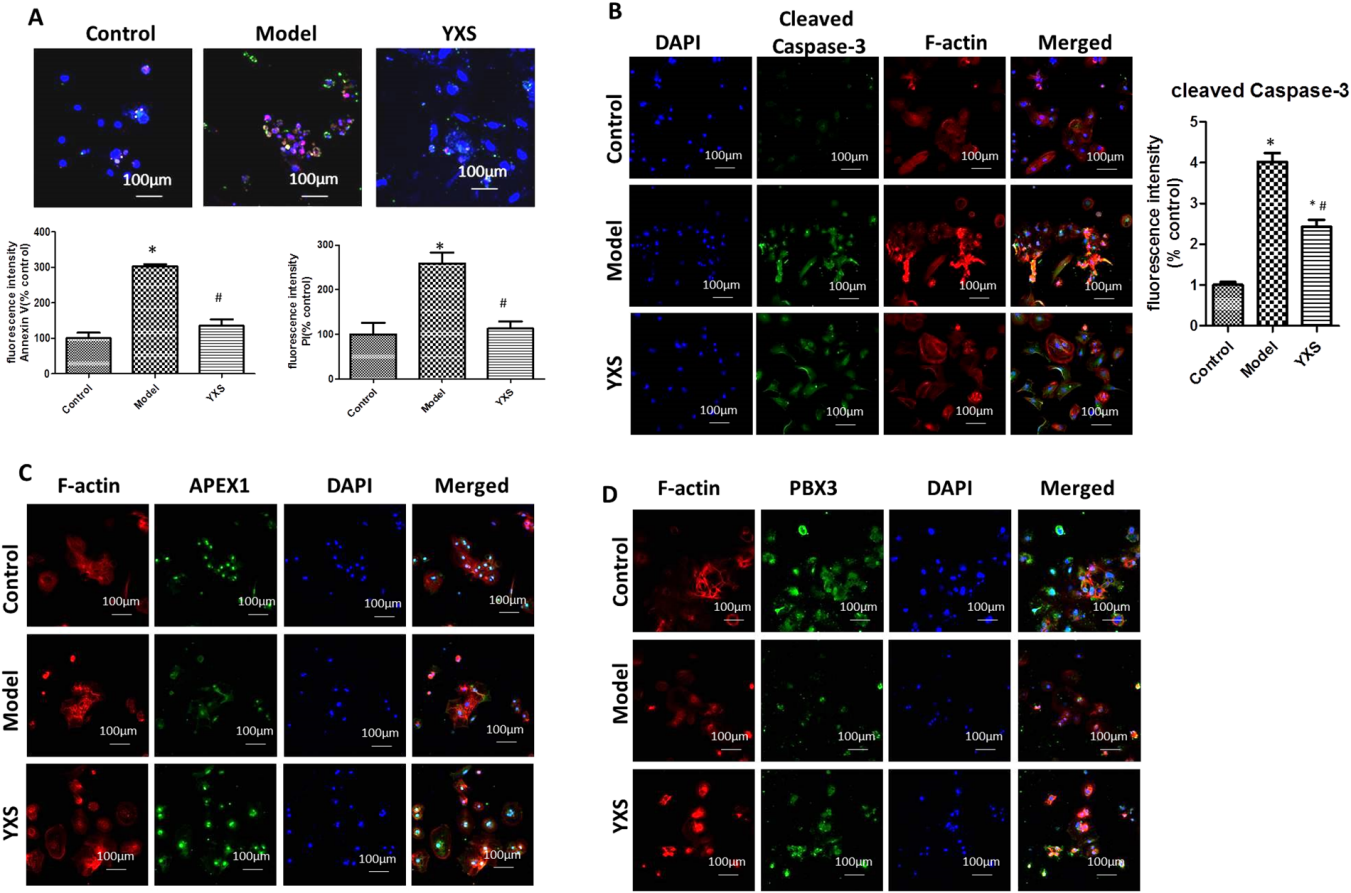
The pharmacological effects of YXS and the activity of TFs were confirmed in the hiPS-CM cell model. (**A**) YXS decreased cell apoptosis, as indicated by the decreased staining of both Annexin V (green) and PI (red). The nucleus was stained with DAPI (blue). The number of Annexin V-positive and PI-positive cells was normalized to that of the control group and expressed as a percentage of the control. (**B**) YXS reduced cleaved caspase-3, as indicated by immunofluorescence staining (green). The fluorescence intensity was normalized to that of the control group and shown in a bar graph. Cleaved caspase-3: green, F-actin: red, nucleus: blue. Scale bar: 100 μm. (**C**,**D**) Nuclear translocation of APEX1 and PBX3 was verified in the hiPS-CM cell model, as indicated by immunofluorescence staining of APEX1 and PBX3. APEX1 and PBX3: green; F-actin: red, nucleus: blue. The data are presented as the mean ± SD from three independent experiments. *P < 0.05 compared to the control group; ^#^ *P* < 0.05 compared to the model group. Scale bar: 100 μm.

The effects of YXS against H_2_O_2_-induced damage and the activity of APEX1 and PBX3 were further verified in a hiPS-CM cell model. As shown in Fig. 8A, exposure of hiPS-CM cells to H_2_O_2_ increased Annexin V staining to 303 ± 11% (p < 0.05) and PI staining to 259 ± 47% (p < 0.05) of the control group, whereas YXS treatment significantly reduced the staining to 135 ± 30% and 112 ± 27% of the control, respectively. In addition, increased cleaved caspase-3 staining (4.02 ± 0.6-fold of the control, p < 0.05) was observed after H_2_O_2_ treatment, and this was decreased by YXS (2.31 ± 0.5, 5-fold of the control) (Fig. 8B), indicating that YXS protected hiPS-CM cells against H_2_O_2_-induced damage. Moreover, the decrease in the nuclear staining of APEX1 and PBX3 observed after H_2_O_2_ treatment was reversed by YXS, which confirmed the results from the H9c2 cell model (Fig. 8C,D).

## Discussion

Oxidative stress plays a vital role in the pathogenesis of many complicated diseases such as diabetes, ageing and cardiovascular diseases^1,2^. However, the mechanisms involved in oxidative stress remain unclear. Due to low TF abundance, large-scale quantitative analysis of TFs remains a challenge, and it is important to reveal the underlying mechanisms in regards to the activity of transcription factors in oxidative stress. In this study, an integrated strategy was developed to find critical transcription factors through integrating TFs activity and their downstream genes and then combined with a network pharmacology analysis. By using the integrated strategy, Apex1, Pbx3, and along with other 5 TFs with their functions involved in anti-oxidation, anti-apoptosis and DNA repair might be important transcription factors that mediate YXS’s protection against H_2_O_2_-induced oxidative stress. Moreover, YXS initiated biological process such as anti-apoptosis and DNA repair to protect cardiomyocytes against H_2_O_2_-induced damage. The findings offer novel insights into the underlying mechanisms of YXS for the treatment of heart disease.

**Figure 9:**
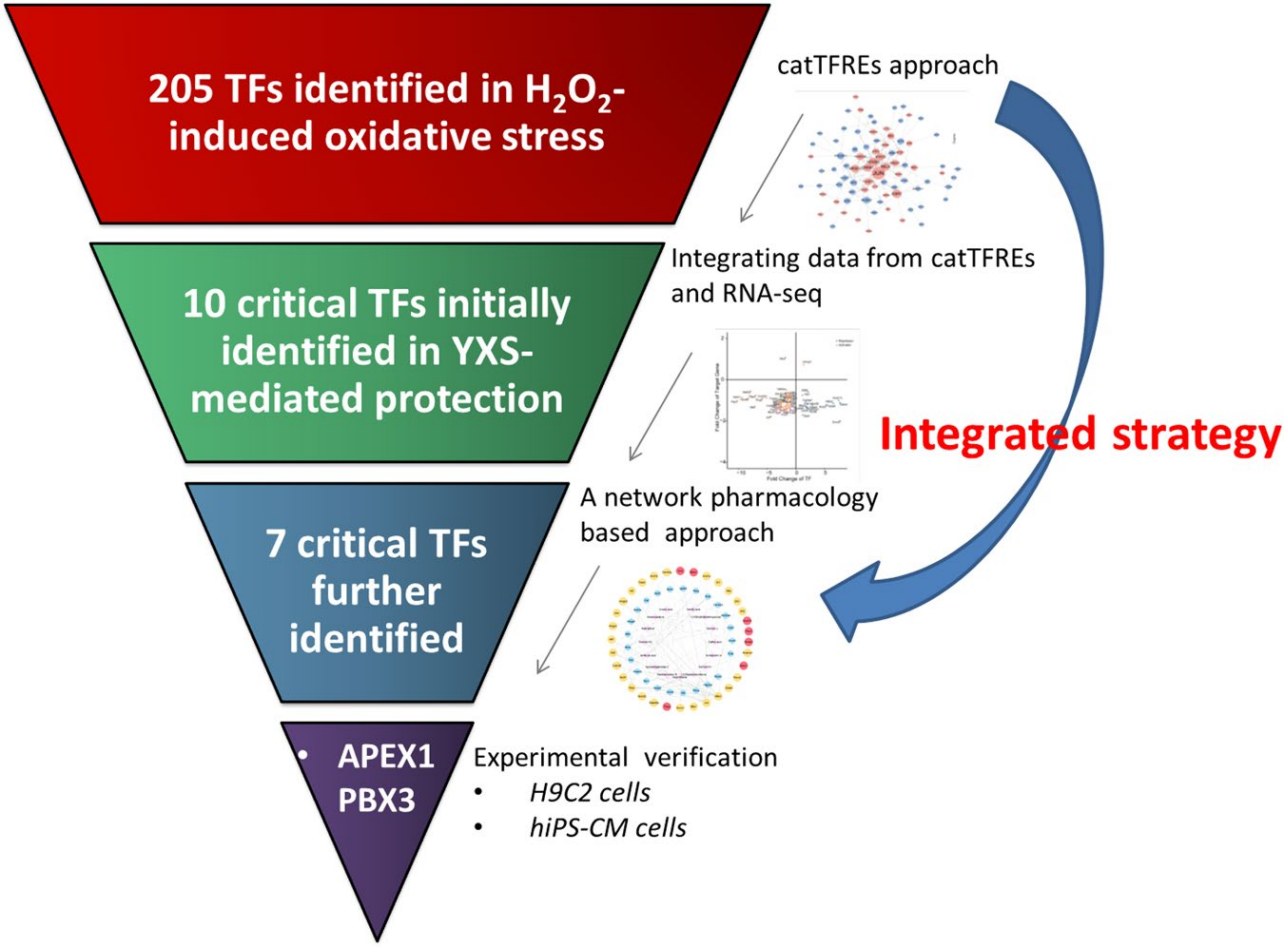
Representative diagram of the integrated strategy used for revealing critical transcription factors in the protection of YXS against H_2_O_2_ -induced oxidative stress.

An integrated strategy was successfully constructed to reveal critical TFs in the protection of YXS against H_2_O_2_-induced injury as shown in Fig. 9. This integrated strategy could reduce the background interference to the minimum and find out the fundamental critical transcription factors. Moreover, considering the binding activity of TFs to a specific DNA sequence and their downstream genes expression levels at the same time could reflect the activity and functions of the transcription factors more accurately. Briefly, 205 TFs have been found through a quantitative profile of TF activity by catTFRE method which was realized by building a DNA construct containing tandem transcription factor DNA response elements with an affinity to specific TFs^15^. Then 10 critical TFs were initially screened out by integrating TFs activity and their downstream genes based on their gene expression and regulatory directions. Integrating the data from the RNA-seq transcriptome and catTFRE proteome could reduce the background interference to the minimum and find out the fundamental critical transcription factors in complicated biological process. Finally, 7 critical TFs were further identified through a network of chemical components of YXS and their potential targets via a network pharmacology analysis. Need to notice, up-regulation of Apex1 could inhibit oxidative stress and enhanced antioxidant ability through regulating Nrf2 which was consistent with our result^29,31^. Meis1 deficiency resulted in increased oxidative stress and cell apoptosis^32^. Arnt could reduce ROS levels, and the deficiency of Arnt in the mouse hearts resulted in cardiomyopathy^33^. All these findings indicated that the integrated strategy was effective to discover critical transcription factors in the protection of YXS against H_2_O_2_-induced oxidative stress.

Cell apoptosis and DNA repair were important in YXS-mediated protection against H_2_O_2_-induced injury. ROS play vital roles in oxidative stress-mediated cardiac injury, as they can attack biological molecules such as lipids, proteins, nucleic acids and enzymes and induce a series of damaging processes including DNA crosslinking, enzyme deactivation, and protein degradation, resulting in eventual cell death. Among the responses, cell apoptosis was a typical phenomenon that occurs in response to oxidative stress. In this study, typical apoptotic characteristics of cardiomyocytes were observed, such as increased cleaved casapase-3 and an increase in the loss of mitochondria membrane potential, and these outcomes were obviously rescued by YXS (Fig. 1). Notably, the apoptosis-related TFs had a significantly larger network degree than the non-apoptosis-related TFs, indicating the potent ability of YXS in cell apoptosis resistance (Fig. 2). For instance, the transcriptional repression of many anti-apoptotic TFs upon H_2_O_2_ treatment could be reversed after YXS treatment (Table S1). For example, homeobox protein SIX1 (Six1) can inhibit apoptosis by decreasing caspase-3 expression^6^, and Forkhead box protein C1 (Foxc1) enhances apoptosis resistance during oxidative stress by targeting FOXO1^34^. Among identified seven TFs, anti-apoptotic TFs such as Apex1^28,35^, Pbx3^36,37^, Tfcp2^38^, and Arnt^33^ were obviously repressed, and their target anti-apoptotic genes such as Asxl1^39^, Bcl9^40^, Fat1^41^ and Arhgef12^42^ were also down-regulated, indicating that cell apoptosis occurred at the molecular level after H_2_O_2_ treatment. By contrast, YXS activated anti-apoptotic TFs such as Apex1, Pbx3, Tfcp2, and Arnt, and increased anti-apoptosis genes such as Asxl1, Arhgef12^42^ and Cenpf^43^, demonstrating a vital protective role in H_2_O_2_-induced injury. Moreover, the DNA repair process was also activated by YXS, as indicated by the results from the RNA-seq data (Fig. 2B). DNA damage could be generated by the high activity of •OH generated from H_2_O_2_ decomposition^44,45^. Notably, the ROS-induced DNA damage could in turn cause the generation of more ROS, resulting in a malignant injury cycle^46^. The analysis of genes downstream of TFs further demonstrated that YXS strongly activated the DNA repair system, as indicated by the elevated expression of DNA repair-related genes, such as Pen1, Pole, Eme1, Ung^47^, Dtl^48^, Ssrp1 and Mcm10^49^. Taken together, these results indicated YXS activated biological processes such as anti-apoptotic process and DNA repair to prevent H_2_O_2_-induced damage.

In this study, Apex1 and Pbx3 were identified as critical TFs in the protection of YXS against H_2_O_2_-induced injury from the results of the integrated strategy and subsequent validation experiments. Apex1 plays a central role in oxidative stress, as its major role was involved in DNA repair and cell redox homeostasis regulation^28,29^. To initiate DNA repair, Apex1 catalyses the hydrolytic cleavage of the phosphodiester backbone adjacent to the injury^50^. Overexpressed Apex1 could reverse O_2_- production and cell apoptosis in cardiomyocytes both *in vitro* and in myocardial ischaemia-reperfusion^35^. Similarly, our result showed that increased cell apoptosis was associated with suppressed Apex1 activity in the H_2_O_2_-treated group and the reduced apoptosis was accompanied with increased Apex1 activity after YXS treatment. The analysis of Apex1 target genes also indicated that Apex1 played a role in anti-apoptosis and DNA repair as evidenced by decreased anti-apoptotic genes expression such as Asxl1^39^ after H_2_O_2_ treatment and up-regulated DNA repair-related genes, including Pen1, Pole, Ung^47^, Eme1, Dtl^48^, Ssrp1, and Mcm10^49^ after YXS treatment. The elevated gene expression of Mcm2, Mcm5, and Mcm7 and Mcm 10 after YXS treatment indicated enhanced DNA damage resistance as Mcm10 acts as a replication initiation factor that brings together the MCM2-7 helicase to initiate DNA replication and reduce DNA damage^49,51^. Pbx proteins are a family of TALE (three amino acid loop extension) class homeodomain transcription factors consisting of Pbx-1, 2, 3, 4, which function as Hox cofactors in developmental gene expression^30^. Recent studies revealed that Pbx3 could serve as a cofactor of HOXA9 to inhibit leukaemic cell apoptosis in leukaemogenesis^36,37^. To further reveal the role of Pbx3, its downstream genes were extensively analysed. The pro-survival genes Bcl9^40^, Fat1^41^ and Arhgef12^42^ were all significantly decreased, indicating that serious damage was caused by H_2_O_2_. Importantly, an increase in anti-apoptotic genes such as Arhgef12^42^, and Cenpf^43^ and a decrease in pro-apoptotic genes such as Pbx1^52^ was observed after YXS treatment, suggesting a potential protective role of Pbx3 in H_2_O_2_-induced injury. Thus, Pbx3 might played a role in anti-apoptotic effect as indicated by the increased anti-apoptotic genes expression such as Arhgef12 and Cenpf and decreased pro-apoptotic genes such as Pbx1 after YXS treatment.

Apart from Apex1 and Pbx3, five other TFs, alpha-globin transcription factor CP2 (Tfcp2), homeobox protein Hox-D13 (Hoxd13), DNA methyltransferase 1-associated protein 1 (Dmap1), aryl hydrocarbon receptor nuclear translocator (Arnt), and homeobox protein Meis1 (Meis1) were also identified as important TFs in the YXS-mediated protection against H_2_O_2_-induced injury. Alpha-globin transcription factor CP2 (Tfcp2), also known as LSF, belongs to the LSF/CP2 family, which is related to the Grainyhead family of proteins, and the activity of Tfcp2 is essential for cell cycle progression^53,54^. The transcriptional activity of Tfcp2 is regulated through phosphorylation by both Erk and cyclin C/Cdk2 during G1 phase. Phosphorylation by Erk on Ser-291 and by cyclin C/Cdk2 on Ser-309 results in Tfcp2 suppression, and the dephosphorylation of both sites activates Tfcp2 and facilitates the transition of cells into late G1 phase, prior to the activation of Tyms at the G1/S transition^55,56^. Recent studies have indicated that the suppression of Tfcp2 transcriptional activity results in either apoptosis during S phase or cell cycle arrest at the G1/S transition by down-regulating thymidylate synthase (Tyms)^38^. Dnmt1-associated protein 1 (Dmap1), a component of the NuA4 histone acetyltransferase complex, plays a role in DNA repair and the maintenance of genome integrity^57,58^, and Dmap1 loss results in DNA damage and causes growth arrest due to the activation of cell cycle checkpoints via p53 in mouse embryonic fibroblasts^58^. Meis1, which is a member of the 3-amino-acid loop extension homeodomain-containing family, acts as a vital regulator in the development of the cardiovascular and haematopoietic system^32,59^. Recent studies have indicated that Meis1 controls ROS production via Hif-1α and Hif-2α^59^ and increases oxidative stress, and apoptosis is caused by Meis1 loss^32^. Aryl hydrocarbon receptor nuclear translocator (Arnt), a member of the basic helix-loop-helix Per-ARNT-Sim family, binds to hypoxia response elements (HREs) and HIF1/2α for xenobiotic and hypoxic responses^60^. Arnt has been shown to demonstrate an antioxidant effect by reducing ROS levels, and the deletion of Arnt in the hearts of adult mice resulted in cardiomyopathy^33^. As a member of the HOXD gene family, HOXD13 plays a critical role in tumour development and progression in cancers such as breast cancer and melanoma^61^, and a mutation in HOXD13 was shown to lead to synpolydactyly^62^.

## Conclusion

In this study, an integrated strategy which integrated RNA-seq-based transcriptomics techniques and a newly developed concatenated tandem array of consensus TF response elements (catTFREs)-based proteomics approach and then combined with a network pharmacology analysis, and this integrated strategy was used to investigate critical TFs in the protection of YXS against H_2_O_2_-induced damage in cardiomyocytes. The high-throughput and deep coverage characteristics of this integrated strategy allows it reveal the fundamental alterations occurring and decrease the background interference observed in these types of analyses. YXS initiated biological process such as anti-apoptosis and DNA repair to protect cardiomyocytes against H_2_O_2_-induced damage. By using this integrated strategy, DNA-(apurinic or apyrimidinic site) lyase (Apex1), pre B-cell leukemia transcription factor 3 (Pbx3), and five other TFs were identified as important TFs with their functions involved in anti-oxidation, anti-apoptosis and DNA repair. This study offers a new understanding of the mechanism of YXS-mediated protection against oxidative stress in cardiomyocytes and reveals novel targets for related diseases.

## Electronic supplementary material

Supplementary materials

Supplementary TableS1

Supplementary TableS2
