# Author Correction: Screening and identification of critical transcription factors involved in the protection of cardiomyocytes against hydrogen peroxide-induced damage by Yixin-shu

**DOI:** 10.1038/s41598-018-27137-2

**Published:** 2018-06-07

**Authors:** Jingjing Zhang, Ya Geng, Feifei Guo, Fangbo Zhang, Mingwei Liu, Lei Song, Yuexiang Ma, Defeng Li, Yi Zhang, Haiyu Xu, Hongjun Yang

**Affiliations:** 1 Institute of Chinese Materia Medica, China Academy of Chinese Medical Sciences, Beijing, 100700 China; 2 College of Traditional Chinese Medicine, Shandong University of Traditional Chinese Medicine, Jinan, 250355 China; 3 State Key Laboratory of Proteomics, Beijing Proteome Research Center, Beijing Institute of Radiation Medicine, Beijing, 102206 China; 4 College of Traditional Chinese Medicine, Shandong University of Traditional Chinese Medicine, Jinan, 250355 China

Correction to: *Scientific Reports* 10.1038/s41598-017-10131-5, published online 24 October 2017

This Article contains errors. Additionally, some of the data for control and model samples presented in this study was previously reported by the authors in reference 1,which is not cited in the Article.

The authors performed simultaneous experiments on four experimental groups: control samples, model samples, samples treated with YXS, and samples treated with DHI. These results for YXS and DHI are reported separately in this Article and reference 1, respectively. Some of the data for control and model samples was used both in this Article and reference 1.

Results for the model samples presented in Figure 2A appear in reference 1 as Figure 3A; data for model and control samples shown in Figure 8A and 8B appear in reference 1 as Figure 9B and 9C, respectively. Additionally, a schematic shown in Figure 4A appears in reference 1 as Figure 5A.

Therefore the legend of Figure 2 should read:

“Figure 2. The large-scale quantitative profiling of transcription factor (TF) activity in H9c2 cells in response to H_2_O_2_-induced oxidative stress. (A) The molecular functions of the significantly altered TFs (P < 0.05) are indicated with yellow dots, and the blue dots represent TFs that showed no differences. Model vs control graph republished from [1] with permission. (B) TF regulatory network and boxplots of the network degree for apoptotic TFs and non-apoptotic TFs in the regulatory network.”

The legend of Figure 4 should read:

“Figure 4. The correlation between critical TFs and their target genes in response to H_2_O_2_-induced oxidative stress, based on the direction of TF regulation and the expression level of TFs’ target genes. Yellow nodes refer to activators; blue nodes refer to repressors. Panel (A) reprinted from [1] with permission.”

The legend of Figure 8 should read:

“The pharmacological effects of YXS and the activity of TFs were confirmed in the hiPS-CM cell model. (A) YXS decreased cell apoptosis, as indicated by the decreased staining of both Annexin V (green) and PI (red). The nucleus was stained with DAPI (blue). The number of Annexin V-positive and PI-positive cells was normalized to that of the control group and expressed as a percentage of the control. (B) YXS reduced cleaved caspase-3, as indicated by immunofluorescence staining (green). The fluorescence intensity was normalized to that of the control group and shown in a bar graph. Cleaved caspase-3: green, F-actin: red, nucleus: blue. Scale bar: 100 μm. (C,D) Nuclear translocation of APEX1 and PBX3 was verified in the hiPS-CM cell model, as indicated by immunofluorescence staining of APEX1 and PBX3. APEX1 and PBX3: green; F-actin: red, nucleus: blue. The data are presented as the mean ± SD from three independent experiments. *P < 0.05 compared to the control group; ^#^P < 0.05 compared to the model group. Scale bar: 100 μm. Model and control data in (A) and (B) reprinted from [1] with permission.”

Additionally, in Results section the sentence:

“In addition, increased cleaved caspase-3 staining (4.02 ± 0.6-fold of the control, p < 0.05) was observed after H_2_O_2_ treatment, and this was decreased by YXS (2.31 ± 0.5, 5-fold of the control) (Fig. 8B), indicating that YXS protected hiPS-CM cells against H_2_O_2_-induced damage.”

should read:

“In addition, increased cleaved caspase-3 staining (4.02 ± 0.62-fold of the control, p < 0.05) was observed after H_2_O_2_ treatment, and this was decreased by YXS (2.42 ± 0.84-fold of the control) (Fig. 8B), indicating that YXS protected hiPS-CM cells against H_2_O_2_-induced damage.”

Conclusions of the Article remain unchanged. The authors apologize for the errors.
