# Supplementary material for: Screening and identification of critical transcription factors involved in the protection of cardiomyocytes against hydrogen peroxide-induced damage by Yixin-shu
Source: Sci Rep. 2017 Oct 24;7:13867. doi: 10.1038/s41598-017-10131-5 (PMC5655617; doi:10.1038/s41598-017-10131-5)
Supplement: Supplementary file 1 — Supplementary materials [file 41598_2017_10131_MOESM1_ESM.doc]

**Supplementary Materials**

**Screening and identification of critical transcription factors involved in the protection of cardiomyocytes against hydrogen peroxide-induced damage by Yixin-shu**

Jingjing Zhang^1#^, Ya Geng^2#^, Feifei Guo^1#^, Fangbo Zhang^1^ , Mingwei Liu^3^ , Lei Song^3^ , Yuexiang Ma^4^ , Defeng Li^1^ , Yi Zhang^1^ , Haiyu Xu^1 *^ and Yongjun Yang^1 *^

1 Institute of Chinese Materia Medica, China Academy of Chinese Medical Sciences, Beijing 100700, China.

2 Graduate student of School of Traditional Chinese Medicine, Shandong University of Traditional Chinese Medicine, Jinan, 250355, China

3 State Key Laboratory of Proteomics, Beijing Proteome Research Center, Beijing Institute of Radiation Medicine, Beijing 102206, China.

4 College of Traditional Chinese Medicine, Shandong University of Traditional Chinese Medicine, Jinan, 250355, China

# These authors contributed equally to this work.

*Correspondence and requests for materials should be addressed to HongjunYang (email: hongjun0420@vip.sina.com) and Haiyu Xu (email: hy_xu627@163.com)

**Legends of supplementary table**

**Supplementary TableS1.** Large-scale quantitative profiling of transcription factor (TF) activity of H9c2 cells in response to H2O2 by catTFREs method. And the data were analysed using an intensity-based absolute quantification (iBAQ) approach.

**Supplementary TableS2.** Whole-genome transcriptome profiling of gene expression of H9c2 cells in response to H2O2 by RNA-seq technology. Three biological replicates were used for the RNA-seq experiment.
